# Supplementary material for: Effect of Supplementation with Saccharomyces Boulardii on Academic Examination Performance and Related Stress in Healthy Medical Students: A Randomized, Double-Blind, Placebo-Controlled Trial
Source: Nutrients. 2020 May 19;12(5):1469. doi: 10.3390/nu12051469 (PMC7284642; doi:10.3390/nu12051469)
Supplement: Supplementary file 1 [file nutrients-12-01469-s001.zip › Supplementary Material S3.docx]

**Supplementary Material S3**

**Partial validation of Metanephrine Plasma ELISA for the use with saliva samples**

**Materials and Methods**

Metanephrine Plasma ELISA DEE8100 (Demeditec Diagnostics, Kiel, Germany), which was used in the present study to detect salivary metanephrine (MN), is primarily intended for use with plasma samples. In order to adopt the assay to salivary samples, partial validation procedure was performed as recommended by Andreasson et al. (2015) and Jaedicke et al. (2012).

Three healthy volunteers meeting the inclusion and no exclusion criteria of the trial were recruited and written informed consent obtained. The participants performed saliva collection with the same rules as applied to the trial. By chewing a polyethylene swab of Salivette Cortisol (Sarstedt, Numbrecht, Germany) in sitting position, at least 8 mL of saliva was self-collected. Five aliquots were prepared: four of them, each containing 1.2 mL of saliva, were subjected to sample stability testing, whereas one was stored at -20°C until further analyses.

Sample stability

The four aliquots subjected to sample stability testing were:

1. immediately frozen at -20°C
2. stored at 4°C for 24 hours and then frozen at -20°C
3. stored at room temperature for 24 hours and then frozen at -20°C
4. subjected to three freeze-thaw cycles and then frozen at -20°C

After being thawed, all the salivary samples were processed as described in the trial procedure and subjected to ELISA. The concentration of MN in each (2)-(4) storage conditions were compared to that of (1) with the use of one-way repeated measures analysis of variance (ANOVA).

Protocol modification

The literature reports of MN salivary detection, both chromatography-based (Osinga et al., 2016) and immunoassay-based (Stefanescu, 2011), reveal the concentration of MN in saliva in healthy people to be at the lower limit of quantification (LLOQ) of MN as declared by the producer of the assay used in the present trial (15.1 pg/mL). In order to increase sensitivity of the assay, some modifications of the assay was adopted:

1. in the first step of the assay, in which MN is extracted using an ion exchange matrix, a higher volume of saliva was applied (250 μL instead of 200 μL);
2. after MN extraction to the ion exchange matrix, the smaller volume of eluent was added (55 μL instead of 100 μL) to concentrate MN in the eluate.

Both the steps could theoretically increase the ability of the assay to detect MN by 2.27-times, and subsequently lower the LLOQ to 6.6 pg/mL.

To test whether the modified procedure is able to yield such an increased MN detection, the salivary samples (spiked with 72 pg/mL of MN each) were subjected to ELISA according to both modified and standard procedure. The ratios of obtained results with both methods (modified-to-standard) for each salivary sample were compared to the theoretical value of 2.27 with the use of two-tailed one-sample *t*-test.

Recovery

Each salivary sample was divided into four aliquots and spiked with standard MN solutions to the working spike-derived MN concertation of 72, 240 and 720 pg/mL, respectively. The last aliquot was supplemented with corresponding volume of the standard blank solution. The volume of standard MN solutions used as spikes were lower than 10% of the final volume of spiked salivary samples. The aliquots were subjected to the assay. The differences in MN concentration between spiked samples and the samples with blank solution were calculated and divided by the corresponding theoretical spike concentrations. These values were compared to 100% with the use of a two-tailed one-sample *t*-test.

**Results**

Participants

Three healthy non-smoking volunteers, taking neither medications nor dietary supplements (2 female) with a mean age of 27.0±6.9 years, body mass index of 20.0±2.2 kg×m^-2^ and basal pulse rate of 67.3±9.0 min^-1^ were enrolled.

Sample stability

The salivary samples of MN following three freeze-thaw cycles seem to be stable. 24-hour storage of the samples in the temperature higher than -20°C resulted in gradual MN degradation of up to about 20%. Detailed results of the stability testing is presented in the table below.

**Table. Results of the salivary sample stability testing regarding the content of metanephrine**

|  | MN concentration in saliva samples of each participant [pg/mL] | | | Mean fraction of MN concentration in relation to “immediately frozen” sample [%] (95%CI) | *P*-value  for the comparison to “immediately frozen” sample | |
| --- | --- | --- | --- | --- | --- | --- |
|  | #1 | #2 | #3 |  |  |  |
| Immediately frozen  at -20°C | 20.9 | 29.2 | 21.0 | - | - | test for trend:  0.012** |
| 4°C for 24 h  and then frozen at -20°C | 19.2 | 23.6 | 18.1 | 86.3 (72.3-100.2) | 0.099* |  |
| Room temperature for 24 h and then frozen at -20°C | 17.0 | 23.8 | 16.7 | 80.8 (78.5-83.1) | 0.010* |  |
| Three freeze-thaw cycles  and then frozen at -20°C | 25.7 | 23.0 | 20.9 | 100.2 (45.3-155.2) | 0.88* | |

MN – metanephrine

CI – confidence intervals

* one-way repeated measures ANOVA with two time points

** one-way repeated measures ANOVA with three time points

Protocol modification

A modified ELISA protocol was able to detect 2.11 (95%CI: 1.82-2.39) times more MN than the standard protocol. This increase was not significantly different from that of theoretically assumed 2.27 value (*p*=0.14).

Recovery

Salivary samples were able to recover 114% (95%CI: 101%-128%) of the spiked MN. Although the result was found statistically significant (*p*=0.040), its mean value remained in the acceptable range of 80-120% (Andreasson et al., 2015; Jaedicke et al., 2012).

**References**

Andreasson, U., Perret-Liaudet, A., van Waalwijk van Doorn, L.J.C., Blennow, K., Chiasserini, D., Engelborghs, S., Fladby, T., Genc, S., Kruse, N., Kuiperij, H.B., Kulic, L., Lewczuk, P., Mollenhauer, B., Mroczko, B., Parnetti, L., Vanmechelen, E., Verbeek, M.M., Winblad, B., Zetterberg, H., Koel-Simmelink, M., Teunissen, C.E., 2015. A Practical Guide to Immunoassay Method Validation. Front. Neurol. 6. https://doi.org/10.3389/fneur.2015.00179

Jaedicke, K.M., Taylor, J.J., Preshaw, P.M., 2012. Validation and quality control of ELISAs for the use with human saliva samples. J. Immunol. Methods 377, 62–65. https://doi.org/10.1016/j.jim.2012.01.010

Osinga, T.E., van der Horst-Schrivers, A.N., van Faassen, M., Kerstens, M.N., Dullaart, R.P., Pacak, K., Links, T.P., Kema, I.P., 2016. Mass spectrometric quantification of salivary metanephrines – a pilot study in healthy controls. Clin. Biochem. 49, 983–988. https://doi.org/10.1016/j.clinbiochem.2016.02.003

Stefanescu, A.-M., 2011. Salivary free catecholamines metabolites as possbile biochemical markers in pheochromocytoma diagnosis. Acta Endocrinol. 7, 431–439. doi:10.4183/aeb.2011.431.
