# Supplementary material for: Effect of Supplementation with Saccharomyces Boulardii on Academic Examination Performance and Related Stress in Healthy Medical Students: A Randomized, Double-Blind, Placebo-Controlled Trial
Source: Nutrients. 2020 May 19;12(5):1469. doi: 10.3390/nu12051469 (PMC7284642; doi:10.3390/nu12051469)
Supplement: Supplementary file 1 [file nutrients-12-01469-s001.zip › Supplementary Table S1.docx]

**Supplementary Table S1**

**CONSORT 2010 checklist**

| Section | Item number | Checklist item | Text extracted from the manuscript |
| --- | --- | --- | --- |
| **Title and abstract** | | | |
|  | 1a | Identification as a randomised trial in the title | Effect of supplementation with Saccharomyces boulardii on academic examination performance and related stress in healthy medical students: a randomized, double-blind, placebo-controlled trial |
|  | 1b | Structured summary of trial design, methods, results, and conclusions | In recent years, bacterial probiotic dietary supplementation has emerged as a promising way to improve cognition and alleviate stress and anxiety, however, yeast probiotics have not been tested. The aim of the present study was to determine whether 30-day supplementation with Saccharomyces boulardii enhances academic performance under stress and affects stress markers. The trial was retrospectively registered at clinicaltrials.gov (NCT03427515). Healthy medical students were randomized to supplement their diet with Saccharomyces boulardii CNCM I-1079 or placebo before sitting for an academic examination, which served as a model of stress. The grades of a final examination adjusted to subject knowledge tested in non-stressful conditions were used as a primary outcome measure. Psychometrically-evaluated state anxiety, cortisol and metanephrine salivary levels and pulse rate were tested at a non-stressful time point before the intervention, as well as just before the stressor. Fifty enrolled participants (22.6±1.4 years of age, 19 males) completed the trial in the Saccharomyces and placebo arms. Supplementation with Saccharomyces did not significantly modify examination performance, increase in state anxiety, salivary cortisol and metanephrine. However, the intervention resulted in higher increase in pulse rate under stress as compared to placebo by 10.4 (95%CI 4.2-16.6) min−1 (p=0.0018) and the effect positively correlated with increase in salivary metanephrine (Pearson’s r=0.35, 95%CI 0.09-0.58, p=0.012). An intention-to-treat analysis was in line with per-protocol one. In conclusion, supplementation with Saccharomyces boulardii CNCM I-1079 appears largely ineffective in improving academic performance under stress nor alleviating some stress markers, but it seems to increase pulse rate under stress, which may hypothetically reflect enhanced sympathoadrenal activity. |
| **Introduction** | | | |
| Background and objectives | 2a | Scientific background and explanation of rationale | Cognitive abilities and academic achievements are of paramount importance to medical students as they shape professional competence, help succeed in future career and contribute to overall life satisfaction [1,2]. One of the factors that potentially impairs cognitive efficiency and compromise academic performance is excessive stress. Threat response to a stressor, as found in a cold pressor test, may impair memory retrieval [3], which appears crucial to exam passing rate [1]. Indeed, pre-examination distress in medical students was found to impair cognitive function [4] and predict poor academic performance [5–7]. Medical students tend to be more stressed than their peers, and academic examinations represent a significant source of this stress [5,8]. Such distress may not only impair examination performance, but also deteriorate psychosomatic well-being, promote unhealthy behavior [5,7,9] and lead to neuropsychiatric, cardiovascular and other disorders [10,11]. Academic community should provide counselling on stress management and create a friendly learning environment to promote student well-being and ensure highest academic standards [5,8,11].  In recent years, probiotic dietary supplementation has emerged as a promising way to alleviate stress, reduce anxiety and depression [12,13]. Such interventions have also been tested among medical students in a model of pre-examination stress. Consumption of probiotic Lactobacillus casei strain Shirota YIT 9029 by healthy medical students exposed to examination stress resulted in an anxiolytic effect, as expressed by prevention of salivary cortisol increase, but not by psychometric measures [14,15]. In addition, the intervention improved some indicators of sleep quality [16], certain cold-like and gastrointestinal symptoms, among others [14,15], and also affected serotonin metabolism [14]. Similarly, the administration of heat-inactivated Lactobacillus gasseri strain CP2305 to medical students preparing to take an examination improved sleep quality, prevented the increase of salivary cortisol and affected stress-responsive microRNA expression [17]. Importantly, supplementation of a probiotic mixture containing Lactobacillus and Bifidobacterium exhibited stress-related increase in working memory [18], which could hypothetically enhance academic examination performance [1,19].  Although lactic acid and other bacteria tested as anti-anxiety and cognition-improving agents represent the vast majority of probiotic species, some yeast strains of Saccharomyces are also classified as probiotics [20]. Saccharomyces boulardii, the most investigated species of yeast probiotic, has a well-established therapeutic position in gastroenterology [21]. However, its effect on mental health and cognitive function, particularly in examination stress-exposed medical students, has received very little attention until now. Even so, the results documenting the anti-anxiety and neurocognitive effects of probiotic bacteria cannot be easily extrapolated to yeast probiotics due to their substantial biological differences [22,23]. |
|  | 2b | Specific objectives or hypotheses | The primary aim of the present study was to assess whether healthy medical students demonstrate better performance in academic examination when supplementing their diet for 30 days with a yeast probiotic strain Saccharomyces boulardii CNCM I-1079. The secondary aim was to evaluate the effects of this supplementation on pre-examination psychological, physiological and biochemical stress markers. |
|  |  |  | The secondary outcome of stress markers included state anxiety, salivary cortisol, salivary metanephrine and pulse rate. All the measures were assessed in both “basal” and “pre-examination” settings and the change between two time points was examined. |
| **Methods** | | | |
| Trial design | 3a | Description of trial design (such as parallel, factorial) including allocation ratio | The trial was performed in a unicenter, randomized, double-blind, placebo-controlled, parallel-group setting to investigate whether the intervention demonstrated superior results compared to placebo. |
|  |  |  | The study employed a simple randomization procedure in a 1:1:1 ratio. |
|  | 3b | Important changes to methods after trial commencement (such as eligibility criteria), with reasons | The original protocol of the trial included, apart from placebo, two intervention arms: Saccharomyces boulardii and Lactobacillus rhamnosus GG. However, after trial completion, the Lactobacillus product was found to be degraded; the mean number of colony forming units (CFU) in a single dose was found to be 1.1×10^6, i.e. 0.019% of that of declared (95% confidence intervals, CI 1.8×10^3-7.2×10^8). Thereby, the results of Lactobacillus intervention could be questionable. Consequently, the main analysis in the present article reports the effect of Saccharomyces only, whereas the effect of Lactobacillus is reported in Supplementary Material S1. The methods and detailed results of the internal quality control of the products used in the trial are reported in Supplementary Material S2. |
| Participants | 4a | Eligibility criteria for participants | Healthy third-year medical students were recruited on a voluntary basis. Volunteers considered for entry to the study had to meet the following eligibility criteria.  Inclusion criteria:  • being a third-year medical student of the Faculty of Medicine or Faculty of Military Medicine, Medical University of Lodz, Poland  • age 18-30 years  Exclusion criteria:  • formal inability to sit the first attempt of the final examination of Pharmacology  • chronic diseases: neurological, psychiatric, cardiological, gastroenterological, immunological, endocrine or infectious  • state of immunosuppression  • history of hospitalization (up to three months before entrance to the study)  • presence of central venous catheter  • parenteral nutrition  • current pregnancy or intention to become pregnant within three months from the entrance to the study  • current lactation  • allergic reaction (up to three months before entrance to the study)  • hypersensitivity to yeast, maltodextrins, potato starch, magnesium stearate, hypromellose, gelatin, glycerol or titanium dioxide  • body mass index over 30  • chronic medication use (up to three months before entrance to the study; “chronic” was defined from a frequency perspective as “at least 90 days a year on average”; pharmacological contraceptives were not considered “medication” and were allowed in the study)  • systemic antibacterial or antifungal medication use (up to three months before entrance to the study)  • overuse of alcohol (defined according to the Polish standards [25] as ≥20 g and ≥40 g per day for females and males, respectively) or psychoactive substances (up to three months before entrance to the study)  • tobacco smoking - more than 5 cigarettes (or equivalents) a day (up to three months before entrance to the study)  • pro- or prebiotic preparations intake (up to three months before entrance to the study)  • vegan or other atypical diet  • doing professional or extreme sports |
|  | 4b | Settings and locations where the data were collected | Pre-intervention period was initiated at the study inaugural session, during which enrollment of participants occurred and some basal data were collected. At the beginning of the session held in an academic assembly hall... |
|  |  |  | Participants were asked to collect their salivary samples at home in a non-stressful time in pre-intervention period (to assess “basal” level of cortisol and metanephrine). |
|  |  |  | At the end of the intervention period, a day before the final examination in Pharmacology (Day 29) between 16:00 and 17:00, participants collected a second sample of saliva at home (to assess the “pre-examination” level of cortisol and metanephrine)... |
|  |  |  | On the day of the final examination (Day 30), study participants were asked to come to the examination building 45 minutes before the exam started. They deposed their “pre-examination” salivary samples collected a day before together with their study diaries. Afterwards, participants completed “pre-examination” state anxiety test with the suitable part of the STAI, and self-recorded their “pre-examination” pulse rate after at least 10-minute obligatory rest according to the same protocol as previously described. After their final examination completion, study participants completed a final form... |
| Interventions | 5 | The interventions for each group with sufficient details to allow replication, including how and when they were actually administered | Two probiotic dietary supplements commercially available in Poland were purchased:  1. LacidoEnter capsules (Institut Rosell, Montreal, Canada; batch numbers HG09241 and HI17731; expiry date 01/2017 and 03/2017, respectively) containing lyophilized Saccharomyces boulardii CNCM I-1079 in declared amount of 5×109 CFU per dose,  2. Dicoflor 60 capsules (Dicofarm, Rome, Italy; batch numbers SM513 and SM514; expiry date of both batches 03/2017) containing Lactobacillus rhamnosus GG (ATCC 53103) in declared amount of 6×109 CFU per dose.  In order to obtain indistinguishable formulations, the content of the original capsules were transferred to the new gelatin capsules (ACG Associated Capsules, Maharashtra, India) and filled with a mixture of maltodextrins (Pepees, Łomża, Poland) quantum satis. Placebo products were obtained by filling the same capsules with the mixture of maltodextrins only. The formulations were prepared using a manual capsule filling device (Capsunorm; Eprus, Bielsko-Biała, Poland). |
|  |  |  | While depositing a salivary sample in the Department, participant received a product packaging (indistinguishable between different types of intervention) marked with participant code. The packaging contained 30 capsules with an attached instruction to store it at room temperature in a dry and dark place throughout the study and to take one capsule a day, swallowed as a whole in the morning during or after the breakfast, washing down with a glass of water, for 30 days starting on the designated day. |
| Outcomes | 6a | Completely defined pre-specified primary and secondary outcome measures, including how and when they were assessed | The primary outcome of performance in academic examination was determined according to the number of correctly-answered multiple choice questions in the computer-based final examination in Basic Medical Pharmacology (hereinafter referred to as Pharmacology). Selection of this examination was made on the basis of the results of a pre-study test performed before the trial in a group of fourth-year medical students (n=91), who perceived this exam to be one of the most stressful: 11% of respondents described stress and anxiety experienced during this exam as “frightening”, 28% as “considerable”, 35% as “average”, 24% as “slight” and 2% as “almost none”. The examination in Pharmacology was carried out for third-year medical students in the summer semester. The study participants underwent the examination in three rounds held in two different days due to limited number of examination stations. Each round, another set of randomly chosen questions was included. The round was chosen by participants according to their own preferences.  A pre-examination test in Pharmacology was performed a day before the final examination to be used as a comparator for the results of the formal examination. Its result was not considered the formal academic assessment of the subject, therefore, it was assumed to reflect the actual pre-examination subject knowledge with no effect of stress. This test was internet based (SurveyMonkey; SurveyMonkey, San Mateo, CA, USA) and consisted of 30 yes/no questions (one point for each correctly answered). The content of the test was assessed by two independent academic teachers from the Department of Pharmacology and Toxicology, who were not involved in the trial, as highly relevant for the examination in Pharmacology. Two different versions of the test were used, each before a different day when the final examination was held.  The secondary outcome of stress markers included state anxiety, salivary cortisol, salivary metanephrine and pulse rate. All the measures were assessed in both “basal” and “pre-examination” settings and the change between two time points was examined. While state anxiety, a distress indicator [11], is typically monitored in stress research [14,15], and salivary cortisol is a well-established biological marker of stress reaction [28], metanephrine, particularly salivary, is an uncommonly examined stress-responsive parameter. Metanephrine could be proposed as such to monitor sympathoadrenal manifestation, particularly to validate pulse rate changes [29,30]. Metanephrine is released to circulation as a relatively stable and inactive metabolite of catecholamine epinephrine [31], the major enhancer of heart rhythm [32], and the level of metanephrine correlates with respective catecholamine [33]. Unconjugated metanephrine is easily transported over the salivary gland membrane and is detectable in saliva, however, conflicting results regarding plasma-saliva metanephrine correlation have been published [34,35]. |
|  |  |  | Moreover, pulse rate was manually self-recorded for 30 seconds by palpation at the radial artery after at least 10-minute obligatory rest in a sitting position. Instructions on how to measure the pulse rate as well as the counter that measured the required time were projected in the whiteboard. |
|  |  |  | • State-Trait Anxiety Inventory (STAI) [42], Polish version [43], to assess both state and trait anxiety.  As this session was organized mid-semester, which was considered a relatively non-stressful time period, state anxiety and pulse rate were assumed to be “basal” outcome measures, i.e. not affected by major stressful events. |
|  |  |  | After being thawed, the salivary samples were centrifuged at 1,000 g in 4°C for 5 minutes, and the supernatants were transferred to new vials kept on ice. No apparent blood contamination was visible in any of the tested samples. The concentrations of cortisol and metanephrine were determined in salivary supernatants by an enzyme-linked immunosorbent assay (ELISA) with commercially available kits: Cortisol free in Saliva ELISA DES6611 (Demeditec Diagnostics, Kiel, Germany) and Metanephrine Plasma ELISA DEE8100 (Demeditec Diagnostics), respectively. Two technical replicates were run for each sample. BioTek EL ×800 microplate reader (BioTek, Winooski, VN, USA) was used to measure absorbance at 450 nm wavelength with a reference of 630 nm. A four parameter logistic model was used to plot a calibration curve. The concentrations determined to be below the lower limit of quantification (LLOQ) were substituted by LLOQ/2 values [44]. Cortisol level was determined exactly according to the producer’s instructions. The metanephrine assay was modified and partially validated in terms of sample stability, protocol modification, and recovery to adjust to salivary samples [45,46]. Detailed methods and the results of the assay validation are presented in Supplementary Material S3. |
|  |  |  | On the day of the final examination (Day 30), study participants were asked to come to the examination building 45 minutes before the exam started. They deposed their “pre-examination” salivary samples collected a day before together with their study diaries. Afterwards, participants completed “pre-examination” state anxiety test with the suitable part of the STAI, and self-recorded their “pre-examination” pulse rate after at least 10-minute obligatory rest according to the same protocol as previously described. |
|  |  |  | Figure 1. Timeline of the study procedures |
|  | 6b | Any changes to trial outcomes after the trial commenced, with reasons | None performed |
| Sample size | 7a | How sample size was determined | The required minimum sample size was determined with the use of G*Power version 3.1.9.2 [25]. The model of repeated measures analysis of variance (ANOVA) for within-between interaction was used (suitable for secondary outcome assessment). Statistical power was set to 0.8. As the estimation referred to no preliminary results, medium effect size f=0.25 and default correlation among repeated measures r=0.5 was assumed. The minimum total sample size was determined to be 42 in all three arms of the trial. |
|  | 7b | When applicable, explanation of any interim analyses and stopping guidelines | None perfomed |
| Randomisation: | | | |
| Sequence generation | 8a | Method used to generate the random allocation sequence | The participants then drew paper sheets with printed numbers (hereinafter referred to as “participant code”) and were asked to blind all the tests and biological samples throughout the trial with these codes. |
|  |  |  | The participant codes were entered to a spreadsheet in ascending order. Each code was assigned to a computer-generated pseudorandom integer between 1 and 3, each occurring with the same probability (Statistica 12.5; StatSoft, Tulsa, OK, USA). The 1-to-3 numbers were a priori attributed to the Saccharomyces, Lactobacillus or Placebo arm of the trial, respectively. |
|  | 8b | Type of randomisation; details of any restriction (such as blocking and block size) | The study employed a simple randomization procedure in a 1:1:1 ratio. |
| Allocation concealment mechanism | 9 | Mechanism used to implement the random allocation sequence (such as sequentially numbered containers), describing any steps taken to conceal the sequence until interventions were assigned | The 1-to-3 numbers were a priori attributed to the Saccharomyces, Lactobacillus or Placebo arm of the trial, respectively. |
| Implementation | 10 | Who generated the random allocation sequence, who enrolled participants, and who assigned participants to interventions | Allocation of participants to the groups was carried out by a person not being involved in the rest of the clinical trial to assure allocation concealment... |
| Blinding | 11a | If done, who was blinded after assignment to interventions (for example, participants, care providers, those assessing outcomes) and how | The result of the group allocation was known for neither investigators nor participants to keep both groups blinded until completion of the study. |
|  | 11b | If relevant, description of the similarity of interventions | In order to obtain indistinguishable formulations, the content of the original capsules were transferred to the new gelatin capsules... |
|  |  |  | While depositing a salivary sample in the Department, participant received a product packaging (indistinguishable between different types of intervention) marked with participant code. |
|  |  |  | They were also asked a question “which product, in your opinion, Saccharomyces, Lactobacillus or Placebo, did you take?” |
|  |  |  | Study participants could not identify the product that they had taken (Pearson’s χ2(4)=5.70, p=0.22, Table 3), which confirmed appropriate blinding of the participants and indistinguishability of the product formulations. |
| Statistical methods | 12a | Statistical methods used to compare groups for primary and secondary outcomes | The main analysis of outcome measures was performed per protocol (PP), i.e. for those participants who completed the trial (25 and 25 people in the group of Saccharomyces and Placebo, respectively). Additionally, an intention to treat (ITT) sensitivity analysis was performed each time (31 and 29 people in respective groups).  The results of the final examination in Pharmacology were recorded for all the enrolled participants and the ITT analysis was straightforward. However, some the other outcome measures for dropout participants were missing and were filled in using a multiple imputation by chained equation (MICE) procedure under a missing at random assumption about the unobserved data [47]. The primary outcome of performance in academic examination was assessed using one-way block ANOVA with the examination set as a blocking factor. The results of the pre-examination test in Pharmacology held a day before the final examination were optionally included as an adjustment linear factor. |
|  |  |  | Salivary cortisol and pulse rate data was log-transformed to bring the distribution closer to normal. The secondary outcome of state anxiety, salivary cortisol, salivary metanephrine and pulse rate was assessed using two-way repeated measures ANOVA. The between-subjects factor was the group, whereas the within-subjects factor “basal” and “pre-examination” time points. The two-way within-between interaction was evaluated each time. |
|  | 12b | Methods for additional analyses, such as subgroup analyses and adjusted analyses | Student’s t-test, asymptotic Mann Whitney U test, Pearson’s χ2 test, Fisher’s exact test, Kruskal-Wallis H test and Pearson’s r correlation coefficient were used to compare basic sociodemographic and psychometric variables and in some ancillary analyses. |
|  |  |  | Other general linear models were applied in ancillary analyses to adjust for factors of interest (stress reactivity, sex, self-reported consumption of fermented products and basal pulse rate). |
|  |  |  | To explore the relationship between the reported effects, Pearson’s correlation coefficients were determined in the polled study groups. |
|  |  |  | To further explore whether the reported effects are consistent between individuals with different levels of some covariates, PP and ITT analyses (two-way within-between ANOVA interactions) were repeated with adjustment to stress reactivity (defined as a difference in “pre-examination” and “basal” state anxiety), sex, self-reported consumption of fermented products and basal pulse rate. |
|  |  |  | Moreover, the three-way interaction of the within-subjects factor, the study group and a covariate was assessed in PP and ITT analyses to evaluate whether the effect of Saccharomyces differs between individuals with various levels of a covariate. |
| **Results** | | | |
| Participant flow (a diagram is strongly recommended) | 13a | For each group, the numbers of participants who were randomly assigned, received intended treatment, and were analysed for the primary outcome | Enrollment to the trial was carried out on 20 and 27 April 2016 in two equivalent inaugural sessions and resulted in the enrollment of 92 volunteers in total (60 further allocated to the groups of Saccharomyces and Placebo). The pre-intervention period lasted for 21 to 33 days. Intervention started on 17 May (students of the Faculty of Medicine) and 22 May (students of the Faculty of Military Medicine). Participants took their capsules for 30 days until the final examination held on 15 and 20 June, respectively. The fraction of participants lost to follow up in all three study groups was 16/92, i.e. 17% (10/60, i.e. 17%, in the groups of Saccharomyces and Placebo). Additionally, one participant from the group of Placebo exhibited basal and pre-examination salivary cortisol level that appeared outlying (20.4 ng/mL and 88.2 ng/mL, respectively), indicative of sample contamination or disease, and was removed from PP analyses. The study timeline is presented in Figure 1. A detailed analysis of drop-outs in each group and the number of participants retained is presented in the study flow diagram (Figure 2). |
|  | 13b | For each group, losses and exclusions after randomisation, together with reasons |  |
| Recruitment | 14a | Dates defining the periods of recruitment and follow-up | Enrollment to the trial was carried out on 20 and 27 April 2016 in two equivalent inaugural sessions… |
|  |  |  | Intervention started on 17 May (students of the Faculty of Medicine) and 22 May (students of the Faculty of Military Medicine). Participants took their capsules for 30 days until the final examination held on 15 and 20 June, respectively. |
|  | 14b | Why the trial ended or was stopped | Not done |
| Baseline data | 15 | A table showing baseline demographic and clinical characteristics for each group | The enrolled participants (n=92) were 22.6±1.3 years old; all of them were Polish (apart from a missing data on one subject); 37 (40%) were male. Sociodemographic and basal psychometric data of all the participants are presented in Table 1. The results show no significant group differences between the characteristics. |
|  |  |  | Table 1. Basal sociodemographic, psychometric and laboratory characteristics of the participants enrolled to the study. The results include means and standard deviations or absolute and relative frequencies, if not stated otherwise. |
| Numbers analysed | 16 | For each group, number of participants (denominator) included in each analysis and whether the analysis was by original assigned groups | The main analysis of outcome measures was performed per protocol (PP), i.e. for those participants who completed the trial (25 and 25 people in the group of Saccharomyces and Placebo, respectively). Additionally, an intention to treat (ITT) sensitivity analysis was performed each time (31 and 29 people in respective groups). |
|  |  |  | Figure 2. Study flow diagram |
| Outcomes and estimation | 17a | For each primary and secondary outcome, results for each group, and the estimated effect size and its precision (such as 95% confidence interval) | A PP analysis found no significant difference in final examination score between the Saccharomyces and Placebo groups: 41.9 (95%CI 40.1-43.7) vs. 40.1 (95%CI 38.3-42.0), respectively (F(1,46)=1.85, p=0.18). After adjusting for the results of the pre-examination test in Pharmacology held a day before the final examination, the results remained similarly insignificant: 42.0 (95%CI 40.2-43.8) vs. 40.1 (95%CI 38.3-41.9), F(1,45)=2.15, p=0.15 (Figure 3). Also, no significant difference was observed between the Saccharomyces and Placebo groups regarding the scores obtained in the pre-examination test in Pharmacology: 20.1 (95%CI: 18.6-21.6) vs. 20.7 (95%CI: 19.2-22.1), respectively (F(1,47)=0.32, p=0.57). |
|  |  |  | An ITT analysis resulted in a similar outcome, but closer to statistical significance. The comparison of the final examination scores was 42.8 (95%CI 40.9-44.7) vs. 40.5 (95%CI 38.5-42.4), F(1,56)=2.90, p=0.094, while after adjusting for the results of the pre-examination test was 42.8 (95% 41.0-44.6) vs. 40.4 (95%CI 38.5-42.3), F(1,55)=3.27, p=0.076. No significant difference between the Saccharomyces and Placebo groups was found regarding pre-examination test score: 21.0 (95%CI: 19.6-22.5) vs. 21.2 (95%CI 19.7-22.7), respectively (F(1,57)=0.03, p=0.86). |
|  |  |  | State anxiety  A PP analysis found state anxiety to significantly increase from “basal” to “pre-examination” settings in both Saccharomyces (from 35.9 (95%CI 32.1-39.6) points to 52.7 (95%CI 48.5-56.9) points, F(1,24)=39.11, p<0.0001) and Placebo groups (from 37.1 (95%CI 33.2-41.1) points to 53.1 (95%CI 49.1-57.1) points, F(1,24)=86.69, p<0.0001. There was no significant difference between the extent of increase in state anxiety between the Saccharomyces and Placebo groups: 16.8 (95%CI 11.3-22.3) points vs. 16.0 (95%CI 12.4-19.5) points, respectively (two-way interaction, F(1,48)=0.07, p=0.79) (Figure 4A). |
|  |  |  | An ITT analysis resulted in a similar outcome. No significant difference was found between the extent of increase in state anxiety between the Saccharomyces and Placebo groups: 16.2 (95%CI 11.0-21.4) points vs. 16.9 (95%CI 13.3-20.4) points, respectively (two-way interaction, F(1,58)=0.05, p=0.83).  Salivary cortisol  A PP analysis found salivary cortisol level to significantly increase from “basal” to “pre-examination” settings in both Saccharomyces (from 2.22 (95%CI 1.59-3.09) ng/mL to 3.20 (95%CI 2.51-4.08) ng/mL, F(1,24)=5.29, p=0.030) and Placebo groups (from 2.13 (95%CI 1.66-2.74) ng/mL to 3.06 (95%CI 2.24-4.18) ng/mL, F(1,24)=5.03, p=0.034). There was no significant difference between the extent of increase in salivary cortisol level between the Saccharomyces and Placebo groups: 0.98 (95%CI 0.10-1.86) ng/mL vs. 0.93 (95%CI 0.07-1.78) ng/mL, respectively (two-way interaction, F(1,48)<0.01, p=0.98) (Figure 4B).  An ITT analysis resulted in a similar outcome. No significant difference was found between the extent of increase in salivary cortisol level between the Saccharomyces and Placebo groups: 1.55 (95%CI 0.66-2.44) ng/mL vs. 1.04 (95%CI 0.11-1.98) ng/mL, respectively (two-way interaction, F(1,58)=0.97, p=0.33).  While measuring salivary cortisol level with ELISA, the experimentally set intra- and inter-assay coefficient of variations (CVs) were 7.0% and 10.6%, respectively. LLOQ of cortisol reported by the producer was 0.024 ng/mL and none of the analyzed salivary samples fell below this value.  Salivary metanephrine  A PP analysis did not indicate any significant increase in salivary metanephrine level from “basal” to “pre-examination” settings in the Saccharomyces (from 29.7 (95%CI 23.0-36.4) pg/mL to 38.7 (95%CI 27.4-49.9) pg/mL, F(1,24)=3.86, p=0.061) nor Placebo groups (from 33.9 (95%CI 26.3-41.4) pg/mL to 33.5 (95%CI 25.0-42.0), F(1,24)=0.02, p=0.90). There was no significant difference between the extent of change in salivary metanephrine level between the Saccharomyces and Placebo groups: 9.0 (95%CI -0.5 to 18.5) pg/mL vs. -0.4 (95%CI -7.2 to 6.4) pg/mL, respectively (two-way interaction, F(1,48)=2.78, p=0.10) (Figure 4C).  An ITT analysis resulted in a similar outcome. No significant difference was found between Saccharomyces and Placebo groups regarding the change in salivary metanephrine level: 11.0 (95%CI 2.5 to 19.5) pg/mL vs. 1.5 (95%CI -4.8 to 7.8) pg/mL, respectively (two-way interaction, F(1,58)=3.32, p=0.073).  While measuring salivary metanephrine level with ELISA, the experimentally set intra- and inter-assay CVs were 12.3% and 17.8%, respectively, being consistent with assay characteristic reported by the producer. The adjusted LLOQ (see Supplementary Material S3) was 6.6 pg/mL and five (5%) of the analyzed salivary samples fell below this value.  Pulse rate  Pulse rate significantly increased from “basal” to “pre-examination” time points in both Saccharomyces (from 68.9 (95%CI 65.1-72.9) min−1 to 90.3 (95%CI 84.9-96.1) min−1, F(1,24)=74.93, p<0.0001) and Placebo groups (from 72.9 (95%CI 68.4-77.7) min−1 to 83.9 (95%CI 78.2-90.0) min−1, F(1,24)=35.22, p<0.0001) in a PP analysis. However, the extent of pulse rate increase was significantly higher in the group of Saccharomyces than Placebo: 21.4 (95%CI 16.3-26.5) min−1 vs. 11.0 (95%CI 7.2-14.9) min−1, respectively (two-way interaction, F(1,48)=10.91, p=0.0018) (Figure 4D).  An ITT analysis confirmed this result. The extent of pulse rate increase was significantly higher in the group of Saccharomyces than Placebo: 23.2 (95%CI 18.1-28.2) min−1 vs. 11.8 (95%CI 8.1-15.5) min−1, respectively (two-way interaction, F(1,58)=13.20, p=0.0006). |
|  | 17b | For binary outcomes, presentation of both absolute and relative effect sizes is recommended | No binary outcome measures (apart from ancillary analyses) |
| Ancillary analyses | 18 | Results of any other analyses performed, including subgroup analyses and adjusted analyses, distinguishing pre-specified from exploratory | To explore the relationship between the reported effects, Pearson’s correlation coefficients were determined in the polled study groups. The extent of “basal” to “pre-examination” increase in pulse rate was predicted by both the increase in salivary metanephrine (r=0.35, p=0.012) and increase in state anxiety (r=0.29, p=0.040) in a PP analysis (Table 2). An ITT analysis led to the same statistical conclusion. |
|  |  |  | To further explore whether the reported effects are consistent between individuals with different levels of some covariates, PP and ITT analyses (two-way within-between ANOVA interactions) were repeated with adjustment to stress reactivity (defined as a difference in “pre-examination” and “basal” state anxiety), sex, self-reported consumption of fermented products and basal pulse rate. The results were consistent with the unadjusted analyses presented above.  Moreover, the three-way interaction of the within-subjects factor, the study group and a covariate was assessed in PP and ITT analyses to evaluate whether the effect of Saccharomyces differs between individuals with various levels of a covariate. The majority of the results implicated no influence of a covariate on the effects of interest. The only difference was in relation to consumption of fermented products and basal pulse rate: the individuals who reported consuming more fermented products (or having lower basal pulse rate) presented higher increase in pulse rate under stress in response to Saccharomyces supplementation (three-way interaction, F(1,46)=7.40, p=0.0092 in a PP analysis and F(1,56)=4.67, p=0.035 in an ITT analysis, respectively). Moderating effect of basal pulse rate may reflect the same phenomenon as represented by consumption of fermented products. The details of these analyses are reported in Supplementary Table S2 and Supplementary Figure S1.  Analysis of the post-examination survey revealed the following results. Participants who completed the trial admitted good adherence to the study protocol with no significant difference between the study groups (median, 1st-3rd quartiles: 4, 4-5 vs. 4, 4-5 vs. 4.5, 4-5, in the group of Saccharomyces, Lactobacillus and placebo, respectively; Kruskal-Wallis H test, χ2(2)=0.47, p=0.79). Study participants could not identify the product that they had taken (Pearson’s χ2(4)=5.70, p=0.22, Table 3), which confirmed appropriate blinding of the participants and indistinguishability of the product formulations. A total of 57 participants (74%) assessed the trial as “very well” prepared and conducted, whereas the rest of them returned “quite well”. |
|  |  |  | To assess sample representativeness, some characteristics of the enrolled volunteers (n=92) were compared to that of their classmates who decided to not participate in the trial or met any exclusion criteria (n=439). The enrollment rate was not significantly different between the students from Faculty of Medicine and those of the Faculty of Military Medicine (odds ratio 1.50, 95%CI 0.93-2.42, χ2(1)=2.73, p=0.098). The odds of the women enrolled to the trial was also not significant (1.06, 95%CI 0.67-1.68, χ2(1)=0.07, p=0.79). The scores in the final examination in Pharmacology of enrolled students was not significantly different from that of those who were not enrolled: two-way block ANOVA with “faculty” as a blocking factor, mean score difference 1.1, 95%CI -0.4 to 2.7, F(1,528)=2.10, p=0.15). |
|  |  |  | The effect of intervention with Lactobacillus product is reported in Supplementary Material S1. The results of the internal quality control of the products used in the trial are reported in Supplementary Material S2. The results of the partial validation of metanephrine assay for the use with saliva samples are reported in Supplementary Material S3. |
| Harms | 19 | All important harms or unintended effects in each group | During 30-day follow-up, two out of 82 participants who started the intervention (2%) reported an infection that required antibiotic use: one in a group of Saccharomyces and one in Lactobacillus (Fisher’s exact test, p=1.0). These participants were excluded from the trial. According to the study diaries of the participants who completed the trial, 26 participants (34%) reported any AE in the 30-day follow-up period. There was no significant difference between the incidence of any AE between the study groups (Pearson’s χ2(2)=0.05, p=0.97). The reported AEs were:  • cold - experienced by 11 participants (14%) for the median number of days of 3 (1st-3rd quartile: 2-5) in 30-day follow-up; no significant difference in the number of days with cold were reported between the study groups (Kruskal-Wallis H test, χ2(2)=0.82, p=0.66);  • fever - experienced by two participants (3%) for the median number of days of 1.5 (1st-3rd quartile: 1-2) in 30-day follow-up; both cases were in the Lactobacillus group, however, the difference in the number of days with cold were found statistically insignificant between the study groups (Kruskal-Wallis H test, χ2(2)=3.97, p=0.14);  • gastrointestinal symptoms - experienced by 22 participants (29%) for the median number of days of 2 (1st-3rd quartile: 1-3) in 30-day follow-up; no significant difference in the number of days with cold were reported between the study groups (Kruskal-Wallis H test, χ2(2)=0.13, p=0.94).  No other AE were reported. |
| **DIscussion** | | | |
| Limitations | 20 | Trial limitations, addressing sources of potential bias, imprecision, and, if relevant, multiplicity of analyses | Apart from generalizability issues, the present study has some substantial limitations that warrant mention while interpreting the results. Firstly, the methods used to assess sympathoadrenal activity are modestly representative. Self-reported pulse rate may not be accurate enough as discussed above. No tests other than salivary metanephrine were performed to confirm sympathoadrenal effect. Further studies would require other and more objective cardiovascular measures such as electrocardiogram, heart rate variability, blood pressure monitoring, metanephrine together with normetanephrine assessment and possibly other time-points or within-examination monitoring. Secondly, the performed analyses may be underpowered to detect some of the effects as probability values of some statistical tests consistently approach significance level. At this time, however, no conclusion can be drawn from p-values not exceeding significance level. Thirdly, although the self-reported trial protocol adherence was monitored, neither any adherence tracking devices were used nor the stool specimens were evaluated for presence of supplemented yeast. Fourthly, it cannot be expected that the postulated effect lasts long following supplementation completion as Saccharomyces boulardii has ability to survive in healthy human intestines no more than a few days [69]. Fifthly, the results should not be interpreted as a stimulus for pharmacological management of stress, to not induce overmedicalization [74]. Instead, effective coping strategies together with psychological interventions should be encouraged. Sixthly, failure to include an acceptable quality comparator of Lactobacillus intervention leaves the obtained results with no relation to well-investigated anxiolytic effect of bacterial probiotics [12,13]. Lastly, the trial was registered in a public register after its completion, which may be regarded as holding potential publication bias [75]. The authors were not aware of the need of pre-registration at that time, as they confused the national guidelines on registration of clinical trials of medicinal products only [76] with the requirements of the International Committee of Medical Journal Editors [77]. The authors attest that, apart from this important issue, they made every effort to comply with the highest methodological standards. |
| Generalisability | 21 | Generalisability (external validity, applicability) of the trial findings | The present study examined only a particular probiotic strain of Saccharomyces boulardii (CNCM I-1079) [68], and the results cannot be generalized to other strains such as CNCM I-745 [69]. It was postulated that even distinct technological preparations of the same probiotic strain might affect clinical outcome [70]. Regarding the application of academic examination in stress research, Stowell [48] raised some methodological doubts. The present study, however, acceptably fulfills his criteria for an appropriate model of stress. Regarding the sample representativeness, although several exclusion criteria existed in the trial, the sample was consistent with the third-year medical student population of the Medical University of Lodz. Moreover, the study sample presented trait anxiety [71] and depressive features [72] comparable to that of other examined samples of medical students, suggesting its representativeness of the population of medical students. Taking into account some dissimilarity of medical students from the general population of young adults [8,73], no further attempts of generalization are recommended. |
| Interpretation | 22 | Interpretation consistent with results, balancing benefits and harms, and considering other relevant evidence | The study reveals for the first time the effect of 30-day supplementation with a fungal probiotic Saccharomyces boulardii CNCM I-1079 on academic examination performance and related stress in healthy medical students. The supplementation has significant effect on neither performance in academic examination nor state anxiety, salivary cortisol and metanephrine. However, the supplementation appears to increase pulse rate under stress, which may hypothetically reflect enhanced sympathoadrenal activity. The results of pulse rate increase should be regarded with caution due to limited validity of the measure. The findings require confirmation and exploration at clinical, preclinical and in vitro levels, applying more accurate methods. The study has the potential to drive research forward particularly in the neglected field of pro-cognitive and anti-anxiety properties of fungal probiotics. |
| **Other information** | | | |
| Registration | 23 | Registration number and name of trial registry | The study protocol was retrospectively registered in the U.S. National Institutes of Health on 09 February 2018 and is available at https://clinicaltrials.gov under accession number NCT03427515. |
| Protocol | 24 | Where the full trial protocol can be accessed, if available |  |
| Funding | 25 | Sources of funding and other support (such as supply of drugs), role of funders | The paper has been supported by the Medical University of Lodz with the grant for young scientists No. 502-03/5-108-03/502-54-157 (received by M.S.K) and the grant No. 503/6-086-01/503-61-001-19-00 (received by J.S). The funder had no role in study design, data collection, analysis and interpretation, decision to publish, or preparation of the manuscript. |

N/A – not applicable
