# Supplementary material for: Effect of Supplementation with Saccharomyces Boulardii on Academic Examination Performance and Related Stress in Healthy Medical Students: A Randomized, Double-Blind, Placebo-Controlled Trial
Source: Nutrients. 2020 May 19;12(5):1469. doi: 10.3390/nu12051469 (PMC7284642; doi:10.3390/nu12051469)
Supplement: Supplementary file 1 [file nutrients-12-01469-s001.zip › Supplementary Table S2.docx]

**Supplementary Table S2**

**Ancillary analyses for different levels of some covariates**

**Table. Ancillary analyses of the effect of *Saccharomyces* supplementation as compared to placebo on all the outcome measures.** Final examination score, increase in state anxiety, increase in salivary cortisol, increase in salivary metanephrine and increase in pulse rate were included in the analyses. Two types of the result are reported: A) the effect of interest after adjusting for a covariate (two-way within-between interaction) and B) the difference in the effect of interest between individuals with various levels of a covariate (three-way interaction of within-subject × “covariate” × “group”). All the analyses are reported as both *per protocol* and *intention to treat*. The results in grey shaded table cells are the primary and secondary outcome measures with no adjusting for covariates. They are reported here for comparative purposes. Statistically significant results are reported in bold.

| Covariate | Type of the result | Type of the analysis | Effect of interest | | | | |
| --- | --- | --- | --- | --- | --- | --- | --- |
|  |  |  | Examination score* | Increase in state anxiety | Increase in salivary cortisol** | Increase in salivary metanephrine | Increase in pulse rate** |
| None | N/A | PP | *F*(1,45)=2.15, *p=*0.15 | *F*(1,48)=0.07, *p=*0.79 | *F*(1,48)<0.01, *p=*0.98 | *F*(1,48)=2.78, *p=*0.10 | ***F*(1,48)=10.91, *p=*0.0018** |
|  |  | ITT | *F*(1,55)=3.27, *p=*0.076 | *F*(1,58)=0.05, *p=*0.83 | *F*(1,58)=0.97, *p=*0.33 | *F*(1,58)=3.32, *p=*0.073 | ***F*(1,58)=13.20, *p=*0.0006** |
| Stress reactivity*** | A | PP | *F*(1,44)=2.07, *p=*0.16 | N/A | *F*(1,47)<0.01, *p=*0.99 | *F*(1,47)=2.71, *p=*0.11 | ***F*(1,47)=11.20, *p=*0.0016** |
|  |  | ITT | *F*(1,54)=3.23, *p=*0.078 |  | *F*(1,57)=0.97, *p=*0.33 | *F*(1,57)=3.24, *p=*0.077 | ***F*(1,57)=15.86, *p=*0.0002** |
|  | B | PP | *F*(1,43)<0.01, *p=*0.98 |  | *F*(1,46)=0.63, *p=*0.43 | *F*(1,46)=0.57, *p=*0.45 | *F*(1,46)=0.01, *p=*0.90 |
|  |  | ITT | *F*(1,53)<0.01, *p=*0.99 |  | *F*(1,56)=0.30, *p=*0.59 | *F*(1,56)=0.13, *p=*0.72 | *F*(1,56)=0.18, *p=*0.67 |
| Sex | A | PP | *F*(1,44)=2.44, *p=*0.13 | *F*(1,47)=0.02, *p=*0.90 | *F*(1,47)=0.03, *p=*0.87 | *F*(1,47)=1.10, *p=*0.30 | ***F*(1,47)=8.05, *p=*0.0067** |
|  |  | ITT | ***F*(1,54)=4.41, *p=*0.040** | *F*(1,57)=0.11, *p=*0.74 | *F*(1,57)=1.28, *p=*0.26 | *F*(1,57)=2.33, *p=*0.13 | ***F*(1,57)=10.88, *p=*0.0017** |
|  | B | PP | *F*(1,43)=0.74, *p=*0.39 | *F*(1,46)=0.56, *p=*0.46 | *F*(1,46)=0.23, *p=*0.63 | *F*(1,46)=0.02, *p=*0.88 | *F*(1,46)=0.36, *p=*0.55 |
|  |  | ITT | *F*(1,53)<0.01, *p=*1.0 | *F*(1,56)=0.62, *p=*0.43 | *F*(1,56)=0.18, *p=*0.67 | *F*(1,56)=0.21, *p=*0.65 | *F*(1,56)=0.76, *p=*0.39 |
| Consumption of fermented products | A | PP | *F*(1,44)=2.14, *p=*0.15 | *F*(1,47)=0.06, *p=*0.80 | *F*(1,47)=0.01, *p=*0.93 | *F*(1,47)=2.49, *p=*0.12 | ***F*(1,47)=12.39, *p=*0.0010** |
|  |  | ITT | *F*(1,54)=3.41, *p=*0.070 | *F*(1,57)=0.09, *p=*0.77 | *F*(1,57)=0.71, *p=*0.40 | *F*(1,57)=3.08, *p=*0.085 | ***F*(1,57)=13.52, *p=*0.0005** |
|  | B | PP | *F*(1,43)=0.39, *p=*0.53 | *F*(1,46)=0.28, *p=*0.60 | *F*(1,46)=1.82, *p=*0.18 | *F*(1,46)=0.26, *p=*0.61 | ***F*(1,46)=7.40, *p=*0.0092** |
|  |  | ITT | *F*(1,53)=1.12, *p=*0.30 | *F*(1,56)=0.36, *p=*0.55 | *F*(1,56)=1.23, *p=*0.27 | *F*(1,56)=0.49, *p=*0.49 | *F*(1,56)=3.08, *p=*0.085 |
| Basal pulse rate | A | PP | *F*(1,44)=2.27, *p=*0.14 | *F*(1,47)=0.20, *p=*0.66 | *F*(1,47)<0.01, *p=*0.97 | *F*(1,47)=1.96, *p=*0.17 | ***F*(1,47)=8.46, *p=*0.0055** |
|  |  | ITT | *F*(1,54)=3.54, *p=*0.065 | *F*(1,57)=0.07, *p=*0.79 | *F*(1,57)=0.01, *p=*0.91 | *F*(1,57)=2.39, *p=*0.13 | ***F*(1,57)=9.11, *p=*0.0038** |
|  | B | PP | *F*(1,43)<0.01, *p=*1.0 | *F*(1,46)=0.31, *p=*0.58 | *F*(1,46)=1.39, *p=*0.24 | *F*(1,46)<0.01, *p=*1.0 | *F*(1,46)=2.37, *p=*0.13 |
|  |  | ITT | *F*(1,53)=0.04, *p=*0.85 | *F*(1,56)=0.85, *p=*0.36 | *F*(1,56)=0.75, *p=*0.39 | *F*(1,56)=0.01, *p=*0.91 | ***F*(1,56)=4.67, *p=*0.035** |

* after adjusting for the results of the pre-examination test in Pharmacology held a day before the final examination

** increase between the log-transformed variables

*** defined as the difference between “pre-examination” and “basal” state anxiety

PP – *per protocol* analysis

ITT – *intention to treat* analysis

N/A – not applicable
